# Proteins with Intrinsically Disordered Domains Are Preferentially Recruited to Polyglutamine Aggregates

**DOI:** 10.1371/journal.pone.0136362

**Published:** 2015-08-28

**Authors:** Maggie P. Wear, Dmitry Kryndushkin, Robert O’Meally, Jason L. Sonnenberg, Robert N. Cole, Frank P. Shewmaker

**Affiliations:** 1 Department of Pharmacology, Uniformed Services University of the Heath Sciences, Bethesda, Maryland, 20814, United States of America; 2 Johns Hopkins Mass Spectrometry and Proteomic Facility, Johns Hopkins University, Baltimore, Maryland, 21218, United States of America; 3 Chemistry department, School of Sciences, Stevenson University, Stevenson, Maryland, 21153, United States of America; National Center of Neurology and Psychiatry, JAPAN

## Abstract

Intracellular protein aggregation is the hallmark of several neurodegenerative diseases. Aggregates formed by polyglutamine (polyQ)-expanded proteins, such as Huntingtin, adopt amyloid-like structures that are resistant to denaturation. We used a novel purification strategy to isolate aggregates formed by human Huntingtin N-terminal fragments with expanded polyQ tracts from both yeast and mammalian (PC-12) cells. Using mass spectrometry we identified the protein species that are trapped within these polyQ aggregates. We found that proteins with very long intrinsically-disordered (ID) domains (≥100 amino acids) and RNA-binding proteins were disproportionately recruited into aggregates. The removal of the ID domains from selected proteins was sufficient to eliminate their recruitment into polyQ aggregates. We also observed that several neurodegenerative disease-linked proteins were reproducibly trapped within the polyQ aggregates purified from mammalian cells. Many of these proteins have large ID domains and are found in neuronal inclusions in their respective diseases. Our study indicates that neurodegenerative disease-associated proteins are particularly vulnerable to recruitment into polyQ aggregates via their ID domains. Also, the high frequency of ID domains in RNA-binding proteins may explain why RNA-binding proteins are frequently found in pathological inclusions in various neurodegenerative diseases.

## Introduction

Accumulation of intracellular and extracellular protein aggregates—frequently in the form of amyloid—is a common feature of multiple age-associated human disorders, particularly neurodegenerative diseases [1, 2]. Amyloid is a highly-ordered aggregate that consists of polypeptides arranged in filamentous, beta sheet-rich structures with abundant interlocking hydrogen bonds between sheets [3–6]. Multiple proteins can adopt this architecture [7], and once established, they all share extraordinary resistance to proteolysis, chaotropic agents, detergents, and mechanical breakage [8–12]. Amyloid aggregates—or their oligomeric precursors—are believed to contribute to cellular toxicity through a variety of mechanisms, such as physically disrupting membranes or sequestering essential heterologous proteins [13, 14].

Triplet CAG expansions resulting in polyglutamine (polyQ) tracts are characteristic of at least nine neurodegenerative diseases [15, 16], of which the most common is Huntington disease (HD). Proteins with expanded tracts of polyQ, which are natively unstructured, are predisposed to adopt conformations with amyloid-like properties [17, 18]. When polyQ tracts of the Huntingtin protein (Htt) exceed ~35 glutamines, Htt fragments can form intracellular inclusions [17, 19–21] resulting in impaired Htt function and aberrant protein interactions [22]. These inclusions (or their early precursors) may confer a dominant gain-of-function cellular toxicity [23, 24]. Other studies suggest that aggregation caused by polyQ expansion can result in loss of protein function (and thus pathology), either directly via functional loss of the aggregating species [25], or from the sequestration of proteins that tightly interact with the aggregate [26, 27]. Thus, the proteins that interact with aggregates may mediate pathological mechanisms, either by conferring new cytotoxicity or by contributing to a general loss of function. For these reasons, determining how and why specific proteins interact with polyQ aggregates is important to understanding—and ultimately combating—disease mechanisms.

Recently, we established a mass spectrometry-based approach for non-targeted identification of intracellular amyloid-forming and amyloid-associated proteins [9, 28]. Our method, called TAPI (Technique for Amyloid Purification and Identification), exploits the biophysical characteristics of amyloid, namely detergent resistance and high molecular weight, to isolate amyloid aggregates from cell lysates. Stringent nuclease treatment followed by SDS-gel electrophoresis eliminates non-specific or loosely associated proteins. Thus the TAPI protocol differs from antibody-based pull-down protocols by limiting positive hits to those most tightly associated with amyloid-like aggregates.

In this study we applied the TAPI protocol coupled with mass spectrometry to aggregates formed by polyQ-expanded huntingtin fragments in both yeast (*S*. *cerevisiae*) and mammalian cells (PC-12, rat neuronal precursor). Previously, various proteins have been shown to interact with Htt or Htt fragments [27, 29–33], but our approach was designed to identify proteins that are directly trapped within the amyloid-like polyQ aggregates to determine the types of proteins most prone to irreversible inclusion. We hypothesized that these aggregates would recruit and/or sequester proteins with common biophysical properties. We observed that inclusion into polyQ aggregates was mediated by long intrinsically-disordered (ID) protein domains (≥ 100 amino acids) in two evolutionary divergent cell models. Also, many proteins normally associated with neuronal aggregation in other degenerative diseases (especially amyotrophic lateral sclerosis (ALS)) were disproportionately recruited into polyQ aggregates in mammalian cells. This study expands the emerging connection between ID domains and neurodegenerative disease [34], and demonstrates that long ID domains predispose proteins to be recruited into amyloid-like aggregates.

## Materials and Methods

### Cell Lines and Maintenance

Yeast strains BY4741 (MATa *his3 leu2 met15 ura3* [PIN+] [psi-]) or W303 (MATa *leu2 ade2-1 ura3 can1 trp1 his3 gal*+) were transformed with Gal-Inducible Huntingtin Exon 1 polyQ expansion plasmids (Htt-Q25-GFP or Htt-Q103-GFP) [9, 35]. Genes (or truncated variants) were cloned into pFPS261 and pFPS262, which encode single HA tags in-frame with the multiple cloning site, thus adding c-terminal epitope tags to genes cloned into the Xho1 site. Plasmids pFPS261 (*CEN LEU2 P*_*GAL1*_) and pFPS262 (*2μ LEU2 P*_*GAL1*_) are respectively derivatives of previously-described pH316 [36] and pH317 [37]. Truncated *SGT2-ΔID* is *SGT2Δ300–346*, and truncated *FUS-ΔID* is *FUSΔ1–135*. Human α-synuclein-GFP was expressed from the previously-described plasmid DK258 (*2μ LEU2 P*_*GAL1*_) [38]. All strains were cultured in synthetic defined media with appropriate auxotrophic selection for plasmid maintenance. Protein expression was induced overnight with growth on selective galactose-containing medium.

The PC-12 cell lines were previously described by Wyttenbach et al. [39]. Briefly, the PC12 cells were stably transformed with Doxycycline-inducible GFP-tagged normal Htt-Q23 Exon 1 or expanded Htt-Q74. Cells were cultured on collagen IV (BD Biosciences) coated T-75 flasks and maintained in DMEM with 75 μg/mL hygromycin, 100 U/mL penicillin/streptomycin, 2 mM L-glutamine, 10% heat-inactivated horse serum, 5% Tet-negative fetal bovine serum and 100 μg/mL G418 at 37°C, 10% CO_2_. Culture reagents were obtained from Corning.

### Technique for Amyloid Purification and Identification (TAPI)

The yeast TAPI protocol was performed as previously described [9] with the following alterations: Buffer A- 30mM Tris-HCl pH = 7.5, 5 mM DTT, 40 mM NaCl, 3 mM MgCl_2_, 5% glycerol, 1x Complete protease inhibitors cocktail (Roche), 20 mM NEM (Sigma), 0.5 μl Benzonase nuclease (250 U/ul; Sigma); RNase A (200 μg/ml; Sigma) treatment for 15’ at 4°C prior to ultracentrifugation at 300,000 *g*.

Mammalian TAPI samples were prepared as follows: a cell pellet of at least 1x10^9^ cells (~100μl in volume) were lysed in cold modified 300 μl RIPA buffer (1% Triton X-100, 0.1% SDS, 1% Sodium deoxycholate, 150 mM NaCl, 10 mM Na_3_PO_4_, 50 mM NaF, 5 mM MgCl_2_, 5mM DTT, 5mM Na_3_VO_4_, with 1x protease inhibitors (Roche) and 33 U DNase 1 (Sigma), 3 mg RNase A (Sigma) and 750 U Benzonase (Sigma)), followed with a 10-minute incubation at room temperature and then 20 minutes of mild rotation at 4°C. Lysates were then spun at low speed (5 minutes centrifugation at 100 *g*) and the pellets were subjected to a second lysing in modified RIPA buffer with rotation for 20 minutes at 4°C. Combined supernatants were run through a 30% sucrose gradient by ultracentrifugation (2 hours at 45000 rpm at 4°C with a Beckman SW-50A rotor). Some samples were analyzed at this point to determine the presence of specific proteins in the pellet fraction by western blotting. After ultracentrifugation, the pellet was re-suspended in high SDS buffer (1x TBS, 5 mM DTT, 5 mM EDTA, 2–4% SDS, with 1x protease inhibitors (Roche)) and incubated with gentle mixing for at least 20 minutes at 37°C. Samples were run at 200 volts on an acrylamide gel (Any kD, Bio-Rad) in 10% glycerol with 0.1% bromophenol blue to monitor sample migration. The top 3 millimeters of the wells were excised and frozen. Frozen gel fragments were thawed and resuspended in elution buffer (10mM Tris pH 8.0, 0.4% SDS, 5mM DTT), then mixed and incubated at 99°C for several minutes. Sample volume was reduced by half in a speed-vac and applied to a desalting column (Zeba spin, Thermo Scientific), pre-equilibrated with 25mm triethylammonium bicarbonate (TeABC). The flow-through was analyzed for elution efficiency by western blot prior to digestion.

Samples were digested for mass spectrometry (MS) analysis using a previously described method [40] with minor modifications. Briefly, DTT was added to the sample to obtain a 15 mM solution prior to addition of iodoacetamide to a concentration of 50 mM followed by a 20-minute incubation at 30°C in the dark. Next, 9M urea was added to the sample, which was then filtered (30 kDA amcon 30K spin filter—centrifuged at 16000 x *g* 5–10 min), and washed with 25mM TeABC twice, and finally trypsin digested (5–10μg trypsin) overnight at room temperature. The sample was retrieved from the column by centrifugation (16000 x *g* for 4 min), and washed with 25mM TeABC prior to lyophilization. Lyophilized samples were analyzed by tandem MS/MS by Johns Hopkins Mass Spectrometry and Proteomics Facility.

### Mass Spectrometry Analysis

Samples were run on Q-Exactive (Thermo Scientific) or Orbitrap Velos (Thermo Scientific) at 70,000 resolution for MS and 17,500 for MS2, or 30,000 resolution for MS and 15,000 for MS2 respectively. The data were collected in data dependent mode with the top 15 precursors chosen for MS/MS. The peptides were eluted with a 90 minute gradient at 300 nanoliters per minute after trapping and desalting for 5 minutes at 5 microliters per minute. Peptides were fragmented with a normalized collision energy of 27 for Q-Exactive, and 35 for Orbitrap Velos. Target values were 3E6 ions with 60 millisecond maximum injection time for MS and 5E4 with 250 milliseconds for MS2 for Q-Exactive and 1E6 ions for MS with a 100 millisecond maximum injection time for MS and 5e4 with 300 milliseconds for MS2 for LTQ Orbitrap Velos.

All data were searched using Mascot (v2.6 Matrix Science) through Proteome Discoverer (v1.4 Thermo Scientific). The database for Yeast included Htt-Q25-GFP in addition to the RefSeq 2014 *Saccharomyces cerevisiae* and the database for Rat included GFP-Htt-Q23 in addition to the RefSeq 2014 *Rattus norvegicus*. Variable modifications included oxidation on Met, deamidation on N and Q, and carbamidomethylation on C. Data were searched with a 30 part per million (ppm) tolerance for precursor mass and 0.03 daltons for fragment masses. Data were searched with and without the MS2 processor node which deisotopes the MS2 spectra to the +1 charge state prior to searching. Data were filtered through the Target Decoy PSM Validator.

The resulting data were filtered through Scaffold software for Total Spectra Count at 5% FDR. Criteria for proteins to be defined as associated with Htt-Q74/103 (Htt-PolyQ aggregate) were as follows: 2 or more total spectra, and present in the expanded Htt-polyQ aggregate while absent in the short Htt-polyQ control sample (henceforth termed binary) in at least 2 of 4 samples examined. Thus, the requirement for a protein to be considered positive is that in at least 2 of the matched samples (containing a pair of Q-short and a Q-long samples) performed at the same time on the same instrument, the protein shows ≥2 spectra in the Q-long and not the Q-short. This resulted in 52 Htt-polyQ aggregate associated proteins identified in *Saccharomyces cerevisiae* (Table 1; S1 File) and 91 Htt-polyQ aggregate associated proteins identified in *Rattus norvegicus* (Table 2; S2 File). In the accompanying supplementary files, we also include the proteins that meet a less stringent threshold: present in the Htt-polyQ aggregate sample while absent in the control for at least one sample set (binary) and 2-fold greater spectra number in Htt-Q74/103 (Htt-PolyQ aggregate) than the short Htt-polyQ control in at least one additional sample pair. As a control for the above method of processing, we also used normalized spectral counting (NCS), a label-free quantification method that compares the number of MS/MS spectra assigned to each protein normalized for the total spectral counting among samples [41, 42]. The NCS processing yielded very similar data sets (data not shown).

**Table 1:** Molecular function of proteins associated with Htt-polyQ aggregates as identified by TAPI (N = 52) in *Saccharomyces cerevisiae*.

Functional Category	% of total	Protein Name
Protein Quality Control/ Chaperone	12%	**Apj1**, Bmh1, Def1, Mca1, Sgt2, Ydj1
RNA/DNA Binding	44%	Ccr4, Cyc8, Dhh1, Eap1, Hrp1, Ixr1, Mbf1, Mcm1, Mot3, Nab3, **Nam8**, New1, Nrd1, Pbp1, Pin4, Pop2, **Puf3**, Snf5, Srp54, Taf5, Tup1, Whi3, Ygr250c
Mitochondrial	8%	**Apj1**, **Nam8**, **Puf3**, Ynl208w
Endocytosis, Vessicle & Cytoskeletal Transport	21%	Akl1, Ent1, Ent2, Gts1, Pan1, Scd5, Sla1, Yap1801, Yap1802, Pin3, Sec24
Other	21%	Cbk1, Epo1, Gal2, Mum2, Nup57, Nup100, Nup116, Pgd1, Sml1, Slm1, Ylr177w

Molecular function determined by gene ontology and Saccharomyces Genome Database. Proteins in bold represent those with overlapping cellular functions; totals exceed 100% because of multiple categorizations. Yeast ribosomal proteins Rpl30 and Rps6b were excluded.

**Table 2:** Molecular function of proteins associated with Htt-polyQ aggregates as identified by TAPI (N = 91) in *Rattus norvegicus*.

Functional Category	% of total	Gene/Protein Name
Protein Quality Control/ Chaperone	26%	ADRM1, **CLTC**, DDI2, DNAJA2, DNAJA4, DNAJB1, DNAJC7, **HSP90AA1**, HSPA8, LAP3, **PPIA**, PSMB2, PSMC1, PSMC2, PSMC3, RAD23B, SGTA, SQSTM1, SUMO2, UBQLN2, UBQLN4, UBXN7, USP7, **VPS35**
RNA/DNA binding*	37%	**AARS2**, AKAP81, **ARL6IP4**, **ATP5A1**, ATRX, DDX5, **DYNC1H1**, EIF4G1, EIF4G2, FASN, FUS, GIGYF2, HNRNPA3, HNRNPF, HNRNPH2, HNRNPM, HNRNPU, **HSP90AA1**, MATR3, NONO, NR3C1, PCBP1, **PPIA**, PRPF40A, PRRC2B, RBMS1, SF1, TDP-43, TCERG1, TCF20, TNRC6B, **TUFM**, XRN2, YTHDF1
Mitochondrial	18%	**AARS2**, ACAD9, ACSF2, **ATP5A1**, **CLTC**, ETFB, GLS, HADHA, IDH2, NDUFS7, OGDH, PCK2, PDHB, SUCLG2, **TUFM**
Endocytosis, Vessicle & Cytoskeletal Transport	14%	AAK1, **ARL6IP4**, ASAP1, CLINT1, **CLTC**, CNN2, **DYNC1H1**, MYO1D, NSFL1C, RAB10, SCYL2, TFG, **VPS35**
Other	15%	EP300, GNAO1, KPRP, LDHA, MAGED1, MLF2, PHGDH, PLEKHB2, PPP2R1A, SIK3, SFN, TGM3, THY1, YWHAB

Molecular function assignments were determined by gene ontology, RGD, and NCBI.

* RNA-binding was assigned in some cases on empirical data [99], thus classification does not necessarily imply primary function. Proteins in bold represent those that were placed in multiple categories, thus totals can exceed 100%. Rat ribosomal proteins Rpl6 and Rpl13a were excluded.

### Bioinformatic Analysis

The proteins identified to be associated with polyQ aggregates using TAPI and MS were further characterized by molecular function, Q/N content and intrinsic disorder. Gene Ontology, Saccharomyces Genome Database (SGD) and the Rat Genome Database (RGD) were used to determine the molecular function of each protein [43–45]. Q/N-rich regions were defined as 30 or more Q/N in an 80-amino acid stretch [46]. For the cases in which data were not available, we developed a PERL-based algorithm to examine protein sequences for Q/N-rich regions (S3 File).

Long intrinsically-disordered (ID) domains were determined using the IUPred-L structural prediction algorithm; ID domains were defined as 30 or more amino acids with a disorder score of 0.5 or greater [47]. To approximate the percentage of proteins in the yeast and rat genomes with long ID regions, 100 and 200 proteins respectively (~ twice the size of each sample data set; S1 and S2 Files), were randomly selected using a random number generator in alignment with the full proteomes of yeast (*S*. *cerevisiae*) and Rat (*R*. *norvegicus*) downloaded from uniprot (www.uniprot.org). Domains with intrinsic disorder were evaluated for all proteins by IUPred-L. Chi-Square 2x2 Fisher’s Exact test (Graphpad software) was used to determine if proteins with long ID domains (≥100 amino acids) were significantly enriched in polyQ aggregates. Analysis was also performed to ensure that the TAPI methodology was not biased towards identifying proteins that are abnormally large or abundant. The size (kDa) and cellular abundance (molecules/cell) was determined for each protein in the yeast sample set and compared with the whole proteome (values accumulated from [48–51]; S1 Fig).

### Western Blotting

Western blotting of cell lysates (input) and TAPI-purified samples were used to verify high molecular weight protein aggregation (observed as large species that cannot migrate beyond the top of an acrylamide stacking gel) and confirm that specific proteins are trapped in polyQ aggregates. Standard Western blotting techniques were employed using nitrocellulose or PVDF membranes, which were probed with primary antibodies against the following targets at dilutions of ~1:5000: αGFP (Roche), αHA (Santa Cruz and Sigma), αErk (Santa Cruz sc-93), αFUS (Bethyl), αhnRNPA1 (Cell Signaling), αRAD23B (Protein Tech), αTDP43 (Protein Tech), and αUBQLN2 (Novus Bio- 5f5). Appropriate HRP-conjugated secondary antibodies were used at 1:1000 dilutions, followed by HRP chemiluminescent substrate (Pierce ECL) for visualization.

### Lysate Partitioning

Analysis of proteins (Bmh1p, Def1p, FUS, Ent2p and Sgt2p) entrapped within aggregates was performed by observing the fraction of protein in the total lysate, supernatant or pellet fraction that partitioned to the stacking well of an acrylamide gel under standard SDS electrophoresis conditions. Briefly, yeast lysates were prepared by mechanical breakage using glass beads in TAPI Buffer A (with RNase A). Pellet and supernatant fractions were prepared as described in the TAPI methodology described above. Cellular fractions were subjected to SDS-PAGE using Any kD gels (Bio-Rad) followed by Western Blotting. The effectiveness of the TAPI buffer to eliminate RNA from the aggregates was examined by RNase-Free Agarose gel electrophoresis with and without nuclease treatment (S1B Fig). The FUS protein is prone to degradation following cell lysis, thus denaturing buffer (10 mM Tris, pH 7.5, 8 M urea) was used to visualize protein levels under conditions in which degradation is greatly inhibited. This enables confirmation that the lysates contain equivalent initial amounts of FUS.

### Confocal Microscopy

Confocal microscopy slides were prepared on Poly-L-lysine coated slides with 1x10^6^ cells/spot, fixed with 4% paraformaldehyde in 1x PBS and permeablized with 0.1% Triton X-100 in PBS. Primary antibodies for RAD23B (Bethyl) or FUS (Bethyl) were added at 1:200 dilution, followed by type-specific Alexa Fluor conjugated secondary antibodies (α-rabbit 647 and α-mouse 568, Southern Biotech) at 1:1000 dilution in 1% fetal calf serum in 1x PBS. Sample slides were mounted with DAPI-containing fluoramount (EMS) and viewed on a Zeiss 710 confocal laser scanning microscope and analyzed using Zen software (2009).

### Thioflavin-T Analysis

Thioflavin-T (Th-T; Sigma) fluorescence was used to determine if HttQ103-GFP aggregates are in an amyloid-like form. Purified Sup35-NM fibers (amyloid positive control from previous study [52]), HttQ25-GFP, and HttQ103-GFP samples were treated with 1μg Th-T in 50mM Tris pH 8.0, 50mM NaCl buffer in a black 96-well plate. Samples were analyzed on a BioTek Synergy H1 plate reader using an excitation of 440 nm and emission at 490 nm. To determine if Thioflavin-T absorbance was significantly different between HttQ25-GFP and HttQ103-GFP, a two-tailed T-test analysis was used.

## Results

### Isolation & analysis of polyQ aggregates in yeast

**Fig 1:**
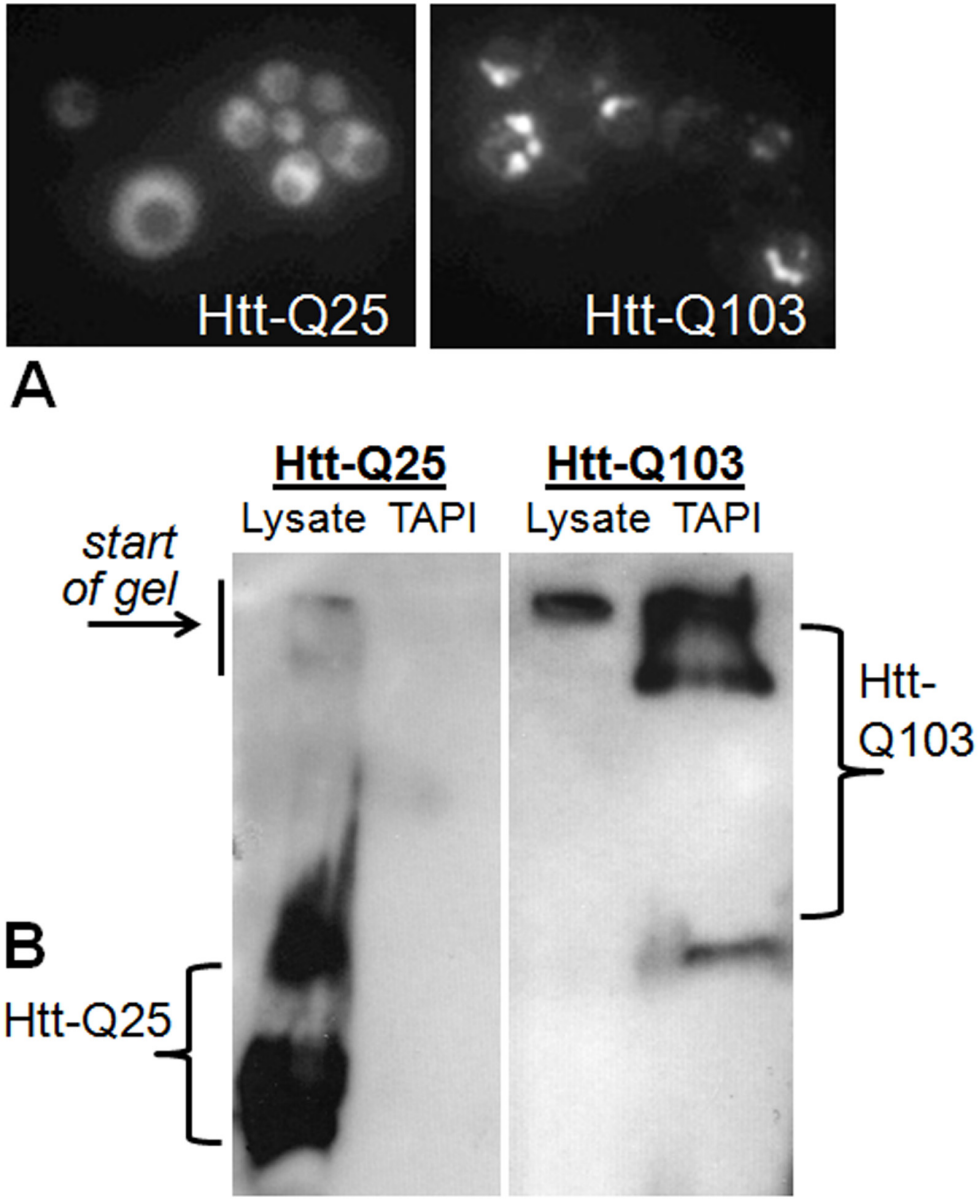
Polyglutamine-expanded Huntingtin exon 1 forms aggregates in yeast that can be isolated by TAPI. (A) Fluorescence microscopy of yeast expressing GFP-tagged Huntingtin exon 1 (Htt) with a normal (Q25) or an expanded polyglutamine tract (Q103). (B) Western blot of GFP-Htt-Q25 and GFP-Htt-Q103 showing that high molecular weight aggregates can be isolated from Htt-Q103 expressing yeast cells. Lysate = input; TAPI = purified aggregate.

To identify aggregate-associated proteins, human HttQ103-GFP and HttQ25-GFP [35] were expressed under control of a galactose-inducible (*GAL1*) promoter in the yeast *Saccharomyces cerevisiae*. Both expression constructs contain the human huntingtin (Htt) exon 1 fragment with polyQ tracts (103 or 25 glutamines, respectively) fused in frame with green fluorescent protein (GFP). As observed previously, HttQ25-GFP is soluble during expression, whereas HttQ103-GFP forms toxic cytoplasmic SDS-resistant aggregates [35] (Fig 1A) that have amyloid-like tinctorial properties and can be trapped at the top of an SDS acrylamide gel [9] (Fig 1B; S1B Fig). Proteins that are specifically associated with Htt amyloid-like aggregates were isolated using the TAPI method [9, 28], which traps the large detergent-resistant species in acrylamide gel matrix for subsequent extraction and identification. As demonstrated in Fig 1B, the TAPI method isolates amyloid-like aggregates of HttQ103-GFP, whereas the non-aggregate-forming HttQ25-GFP does not form species large enough, or sufficiently detergent-resistant, for isolation.

Proteins tightly associated with the isolated Htt-polyQ aggregates were identified using tandem mass spectrometry (MS/MS). Qualitative comparison of all identified proteins from the HttQ103-GFP samples—relative to the HttQ25-GFP samples—reveals a subset that is only associated with the large polyQ aggregates (Table 1; S1 File). In total, 52 proteins were considered polyQ-associated because they were reproducibly found in the expanded HttQ103 aggregate (Table 1) while absent in the HttQ25 control sample (described in methods). To confirm that our approach is not enriching for abundant, large, or charged proteins, the polyQ aggregate-associated proteins were compared against the entire yeast proteome. No obvious differences in size distribution, abundance ([51]; S1 Fig), or charge (the avg. pI of TAPI-identified proteins is 7.1, the same as the approximation for the yeast proteome [53]) were observed between our TAPI-identified proteins and that of the entire yeast proteome.

### Molecular functions of polyQ aggregate-associated proteins in *S*. *cerevisiae*

HttQ103-GFP aggregate-associated proteins were examined using gene ontology (GO) and Saccharomyces genome database (SGD) to assign their functions and properties [43–45, 54]. Unexpectedly, RNA/DNA-binding (mostly RNA binding; Table 1) proteins make up the largest percentage of HttQ103-GFP aggregate-associated proteins. In fact, more than 1/3^rd^ of the polyQ-associated proteins are specifically characterized as RNA-binding proteins (RBPs). Previous studies suggest that RBPs may localize to aggregates because RNA co-aggregates with amyloid-forming proteins [55]. However the TAPI method involves extensive RNase treatment [9, 28]; while we cannot conclude that RNA is completely absent, the vast majority of RNA is eliminated prior to aggregate isolation (S1B Fig). As most RNA-dependent interactions should be lost, a preponderance of RBPs in the HttQ103-GFP aggregate is likely independent of RNA-mediated interactions.

### Yeast proteins recruited into polyQ aggregates share common biophysical properties

PolyQ aggregates have been proposed to induce heterologous protein misfolding via a “cross-seeding” mechanism [56]. Previously, Michelitsch and Weissman observed that Q/N-rich domains have an increased propensity to adapt amyloid structure and concluded that 30 glutamine and/or asparagine residues within an 80-amino acid stretch served as a good predictor of amyloid formation [46]. Assuming that the polyQ aggregates could “cross-seed” such Q/N-rich proteins, we analyzed the 52 identified proteins and found they are significantly more likely to have Q/N-rich domains than the whole of the yeast proteome (54% vs. 2% [46], respectively; S1 File). For comparison, if simply measuring for total glutamine content of the identified proteins, the HttQ103-GFP aggregate-associated proteins have only a two-fold greater total percentage of glutamine content relative to the yeast proteome (respectively, 9.7% vs. 3.8%[57]; S1 File).

**Fig 2:**
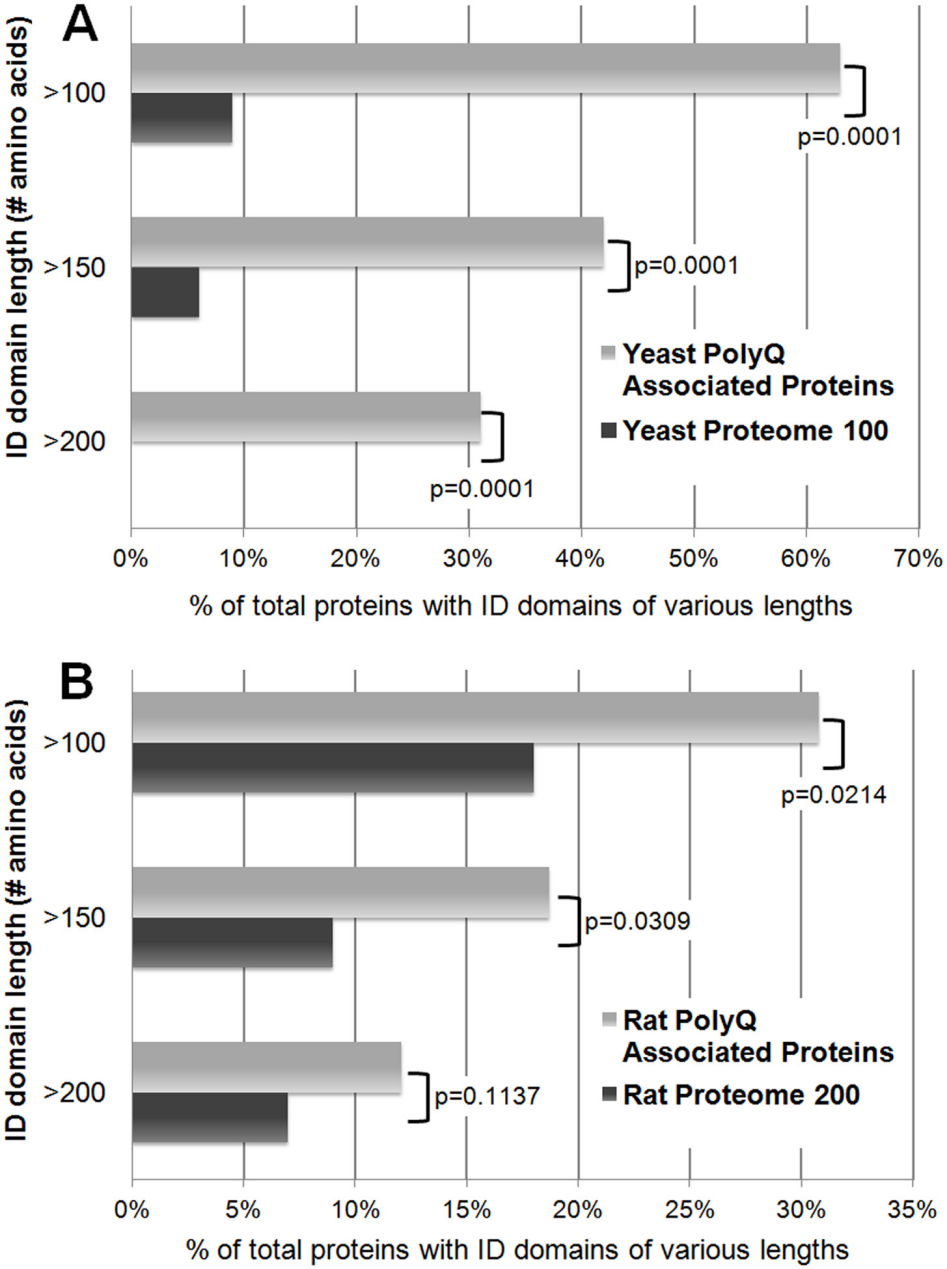
Cellular proteins trapped with Htt polyQ aggregates are disproportionately composed of long intrinsically-disordered (ID) domains. (A) Comparisons of the percentages of proteins with long ID domains of the 52 yeast proteins in Table 1 (reproducibly found by TAPI to be tightly associated with Htt-Q103-GFP aggregates) versus 100 randomly-selected yeast proteins (S1 File). Most of the identified proteins have long ID domains of at least 100 amino acids. (B) Comparisons of the percentages of proteins with long ID domains of the 91 rat proteins in Table 2 (reproducibly found by TAPI to be tightly associated with GFP-Htt-Q74 aggregates) versus 200 randomly-selected rat proteins (S2 File). ID domains are defined as regions of 30 or more amino acids with IUPred scores of 0.5 or greater [47, 98]. Chi-Square Fisher’s Exact test (Graphpad software) was used to determine significance between TAPI-identified proteins and proteome control sets.

An enrichment of Q/N-rich segments also implies an increase in intrinsically unstructured protein domains. When each of the polyQ aggregate-associated proteins was examined for global intrinsic disorder (described here: [58]), the aggregate-associated proteins show a higher average total percent intrinsic disorder relative to the average value for the whole yeast proteome (48% vs. 20%, respectively). However, if the identified proteins are specifically analyzed for containing discrete regions that are intrinsically disordered (using IUPred-L prediction algorithm described in the methods [47]), the polyQ-associated proteins are strongly enriched for the presence of an ID domain (Fig 2). Previous studies have classified ID domains as unstructured regions greater than 20–40 amino acids long [59, 60]. For the yeast proteins associated with polyQ aggregates, almost all have an ID domain of at least ≥30 amino acids in length (92% vs. 31% of the proteome control, respectively; S1 File). However, ~2/3^rds^ of the proteins contain very long ID domains of ≥100 amino acids (63% versus 9%; Fig 2), and strikingly of these proteins, ~1/3^rd^ contain no Q/N-rich domain.

ID domains facilitate protein interactions with RNA, so the large cohort of RBPs we found associated with polyQ aggregates could simply be a result of these proteins disproportionately possessing ID domains. The RBP subset of aggregate-associated yeast proteins was compared to all putative and characterized RBPs in the yeast proteome for ID content (S1 File). A majority (70%) of the HttQ103-GFP aggregate-associated RBPs contain ID domains of ≥100 amino acids, while RBPs in general rarely have such long ID domains (~17%; S1 File). Thus, the high frequency of RBPs in the polyQ aggregates might result from the presence of long ID domains in these proteins, rather than a result of some uncharacterized RNA-binding mechanism of polyQ aggregates.

### Biochemical confirmation of protein recruitment to polyQ aggregates in yeast

Def1p, Ent2p, Sgt2p and Bmh1p are among the proteins we found to be specific to polyQ aggregates in yeast. We also found that mammalian homologs of Ent2p, Sgt2p and Bmh1p (CLINT1, SGTA and YWHAB, respectively) co-aggregate with polyQ in mammalian cells (discussed in detail below). Ent2p, Sgt2p and Bmh1p were previously shown to have effects on protein aggregation (or toxicity) in yeast models [61–63], while Def1p has not been shown to influence protein aggregation. We selected Def1p, Ent2p, Sgt2p and Bmh1p for biochemical confirmation of the MS results.

**Fig 3:**
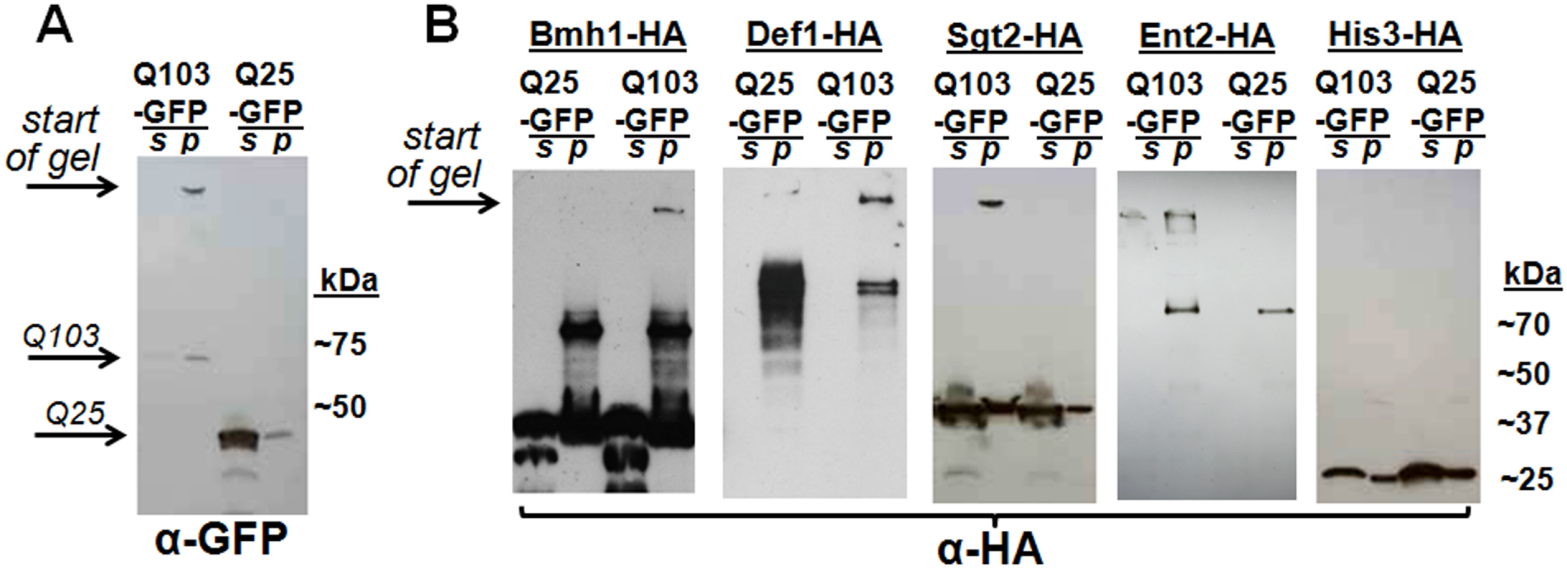
Western blotting confirms that TAPI-identified proteins are trapped in large, detergent-resistant Htt-polyQ aggregates. (A) Immunoblotting reveals that Htt-Q103-GFP, but not Htt-Q25-GFP, forms large detergent-resistant aggregates that fractionate in the pellet (partial TAPI purification; see methods) and remain at the top of an acrylamide gel under SDS-PAGE conditions. (B) Immunoblotting confirms that HA-tagged Bmh1p, Def1p, Ent2p, and Sgt2p (proteins identified by mass spec) get trapped in the detergent-resistant aggregates that can be seen stuck at the top of the gels in the pellet fractions. As a negative control, HA-tagged His3p (not identified by mass spec) shows no susceptibility to co-aggregation with Htt-Q103-GFP. Note that Def1p was not easily visualized in the supernatant fraction because it is prone to degradation (data not shown). Samples were spun at 45,000 rpm, except Ent2p (10,000 rpm). **S** = supernatant; **P** = pellet fraction.

The presence of Def1p, Ent2p, Sgt2p and Bmh1p in polyQ aggregates was tested by immunoblotting following a modified version of our TAPI protocol (Fig 3). HttQ103-GFP, expressed in yeast, forms a high molecular weight aggregate that partitions to the pellet fraction and gets stuck at the top of an SDS-PAGE gel (Fig 3A). When HA-tagged Def1p, Ent2p, Sgt2p and Bmh1p are co-expressed with HttQ103-GFP, they show a similar pattern in their respective western blots, but only when expressed with the long polyQ expansion, not with HttQ25-GFP (Fig 3B). Thus, the interactions of all three proteins with polyQ aggregates are sufficiently strong that they co-fractionate and withstand the conditions of SDS-PAGE, resulting in their retention in the large resistant species that cannot migrate into the gel (Fig 3B). The His3 protein was chosen as a negative control as it was never identified in our TAPI samples. Immunoblotting confirms that unlike Def1p, Sgt2p and Bmh1p, HA-tagged His3p is not entangled within polyQ aggregates (Fig 3B), thus recapitulating our MS results biochemically. To ensure that proteins were not independently forming large detergent-resistant aggregates as a consequence of cellular stress caused by HttQ103-GFP, cells were treated with two alternative stresses: proteasome inhibitor MG132 and over-expressed human α-synuclein protein, which is toxic in yeast cells [64]. Neither condition resulted in Sgt2p getting stuck at the top of an acrylamide gel (S1C Fig).

### Analysis of polyQ aggregate-associated proteins in PC-12 cells

**Fig 4:**
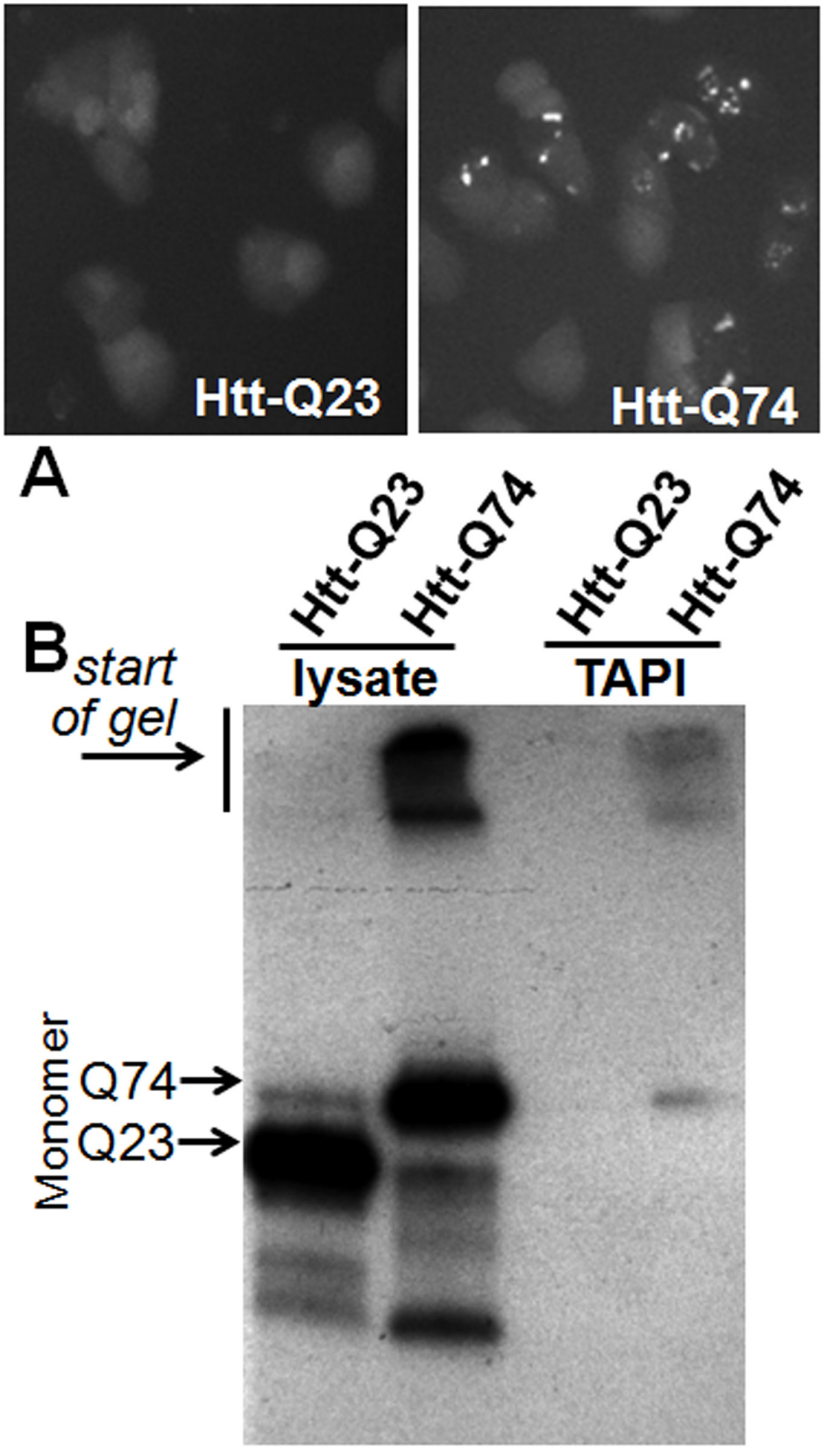
Polyglutamine-expanded Huntingtin exon 1 forms aggregates in PC-12 cells that can be isolated by TAPI. (A) Fluorescence microscopy of PC-12 cells expressing doxycycline inducible transgene GFP-tagged Huntingtin exon 1 (Htt) with normal (Q23) or expanded polygluamine tract (Q74). (B) Western blot of GFP-Htt-Q23 and GFP-Htt-Q74 showing high molecular weight aggregates can be isolated from Htt-Q74 expressing PC-12 cells. Lysate = input; TAPI = purified aggregates.

With the observation that RBPs and proteins with ID or Q/N-rich domains are bound to HttQ103-GFP aggregates in the yeast model, we asked if the same would be true in a mammalian system. To test this, another Huntington disease model was used: mammalian PC-12 cells stably expressing doxycycline-inducible HttQ23-GFP or HttQ74-GFP developed by David Rubinsztein’s lab [39]. Again, both constructs contain the Htt exon 1 fragment with a polyQ tract (23 or 74) fused in frame with GFP, and only the protein with pathogenic extended polyQ forms SDS-resistant aggregates (Fig 4A). In this model, the polyQ aggregates have an approximately equal distribution in the cytoplasm and nucleus [39], thus may interact with most of the non-secreted cellular proteome. The amyloid-forming HttQ74-GFP, but not HttQ23-GFP, could be successfully purified and detected using the TAPI method, as shown in Fig 4B. MS analysis followed by comparison of all identified proteins showed a subset that was unique to samples with polyQ aggregates. Using the criteria described above, 91 proteins were considered specific to the HttQ74-GFP aggregates (Table 2; S2 File).

### Molecular functions of polyQ aggregate-associated proteins from PC-12 cells

Characterization of the proteins enriched in polyQ aggregates from PC-12 cells reveals a disproportionate number of RBPs, as similarly observed in yeast (Table 2; S1 and S2 Files). Also, several functional homologs were common to the polyQ aggregates isolated from both the yeast and mammalian cells (S1 Table): RNA-binding proteins DDX5 (yeast Dhh1p) and hnRNPA3 (yeast Hrp1p), 14-3-3 proteins YWHAB and SFN (yeast Bmh1p), endocytosis proteins CLINT1 (yeast Ent1/2p) and AAK1 (yeast Akl1p), and chaperone proteins SGTA (yeast GET pathway protein Sgt2p), DNAJA2 and DNAJA4 (yeast Ydj1p and Apj1p).

### Biophysical properties of proteins that associate with polyQ aggregates in PC-12 cells

Biophysical characterization reveals the HttQ74-GFP aggregate-associated proteins from PC-12 cells are enriched for Q/N-rich regions relative to the entire rat proteome (7% versus 0.4%), albeit to a much lesser degree than in yeast. Proteins containing long ID domains (≥100 amino acids) are significantly increased among the TAPI-identified HttQ74-GFP aggregate-associated proteins (31% vs. 18% for proteome control, respectively, p = 0.021; Fig 2; S2 File). As in yeast, this suggests that cellular proteins with long ID domains may be inherently prone to inclusion in polyQ aggregates.

### Neurodegenerative disease-linked proteins are recruited into polyQ aggregates

**Fig 5:**
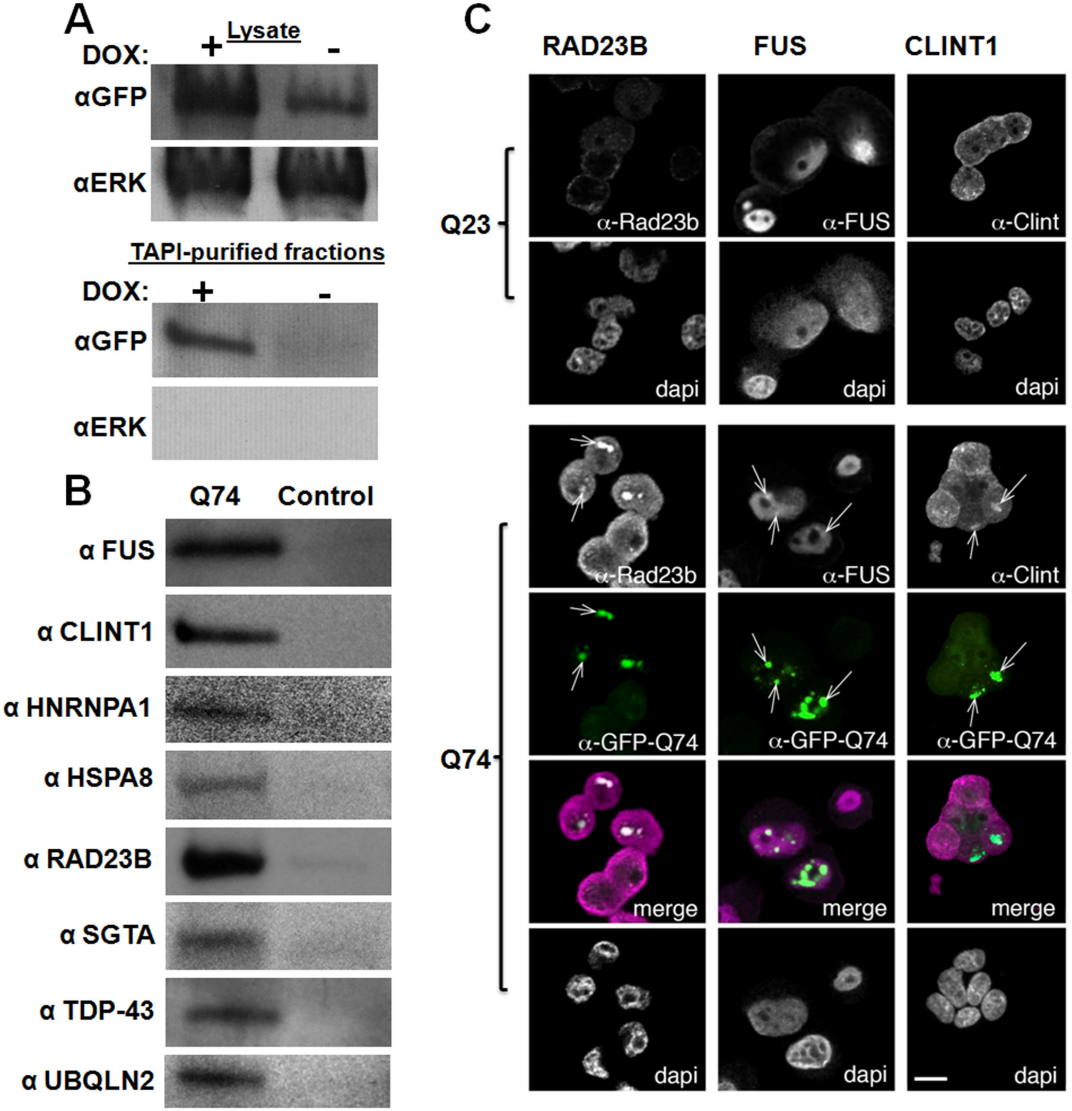
Confirmation of polyQ-associated proteins from PC-12 cells identified by TAPI. (A) Western blotting shows that the addition of doxycycline to the PC-12 cell model induces the expression of HttQ74-GFP, resulting in aggregates that can be purified by TAPI. The kinase ERK is probed as a negative control; ERK was never identified by mass spectrometry, so is not expected to co-fractionate with polyQ aggregates. (B) Western blot analysis of TAPI-purified polyQ aggregates from PC-12 cells confirms the presence of several disease-associated proteins only in the Htt-Q74 samples. All proteins migrated near their predicted molecular weights. For control, the TAPI procedure was conducted in parallel on the induced Htt-Q23 cell line (FUS, TDP-43, UBQLN2, HNRNPA1) or the un-induced Htt-Q74 cell line (CLINT1, HSPA8, RAD23B, SGTA). (C) Confocal microscopy shows localization of identified proteins to Htt-Q74 aggregates in PC12 cells. (left) RAD23B, nominally a DNA repair protein, localizes to nuclear Htt-Q74 inclusions but not cytoplasmic inclusions. (middle) FUS, an RNA-binding protein localizes to nuclear and cytoplasmic Htt-Q74 inclusions. (right) CLINT1, a clatherin-interacting protein, is observed in cytoplasmic Htt-Q74 aggregates. Arrows indicate foci with co-localized proteins. Green = GFP; Magenta = CLINT1, FUS or RAD23B in merge.

Among the aggregate-specific proteins in PC-12 cells, we also identified a significant subset of proteins that are neurodegenerative disease-associated (Table 3). Surprisingly, these proteins were not limited to huntingtin-interacting proteins; we identified a cadre of ALS-linked proteins. We hypothesized polyQ aggregates could pull in proteins that are prone to aggregation in other pathological contexts. When we probed the purified polyQ aggregates (purified fraction confirmed in Fig 5A) with antibodies specific to proteins that are known to aggregate in the motor neurons of ALS patients (and were identified by MS in this study), we corroborated the specific presence of FUS, TDP-43, and UBQLN2 in the Htt-Q74 aggregates (Fig 5B). We hypothesized that other ALS-linked proteins may be similarly recruited into aggregates but escaped detection by MS. Immunoprobing for the ALS-linked HNRPA1 also revealed its presence in the purified polyQ aggregates (Fig 5B). For a control, we probed for the kinase Erk, which was not identified by MS in our samples, and indeed, it could not be found in the polyQ aggregates (Fig 5A). In total, of the HttQ74-GFP aggregate-associated proteins in PC-12 cells, 21% (19/91) have previously been found in the intraneuronal inclusions of various neurodegenerative diseases (Table 3). Of these disease-linked HttQ74-GFP aggregate-associated proteins, many are RBPs (7/19) and more than half (9/19) contain very long ID domains (≥100 amino acids) (Table 3; S2 File).

**Table 3:** Htt-polyQ aggregate-associated proteins found in inclusions and aggregates in various neurodegenerative diseases.

Gene/Protein Name	Disease or Disease Model	ID Domain	RBP	PQC	Reference
AAK1	ALS-SOD1	209, 81, 50			[100]
FUS	ALS	284, 71, 87	*yes*		[101–103]
HNRNPA3	ALS	39	*yes*		[104]
HSPA8	HD & SCA1	46, 36		*yes*	[94, 105]
MATR3	ALS	31, 57, 42, 119, 72	*yes*		[106]
MLF2	HD	136			[107]
NONO	ALS-FUS	66	*yes*		[108]
PPIA	ALS-SOD1	*None*	*yes*	*yes*	[109]
RAD23B	HD, SCA3	120, 67, 41		*yes*	[110, 111]
SQSTM1	PD & ALS	153		*yes*	[112–115]
SUCLG2	AD	*None*			[116]
SUMO2	HD & ALS	*None*		*yes*	[117, 118]
TARBP (TDP-43)	ALS	45, 55	*yes*		[119, 120]
TCERG1	HD	138, 177, 70, 36	*yes*		[74]
TCF20	HD	87, 739, 50, 32, 709, 34, 121	*yes*		[121]
TFG	CMTD	35, 54, 105			[122]
TGM3	HD	49			[123]
UBQLN2	ALS	62, 58, 42, 81, 31, 84		*yes*	[124]
YWHAB	ALS	*None*			[125]

The numbers for ID Domains indicate the length of distinct unstructured regions ≥30, for the identified rat proteins from PC-12 cells, as determined by IUPred-L. RBP = RNA-binding protein as described in the legend of Table 2; PQC = protein quality control.

Since one fifth of the proteins we identified have been previously linked to pathological aggregates, perhaps many of the remaining proteins represent novel aggregate-associated proteins that have never been specifically probed in various pathological contexts. We selected HSPA8, CLINT1 and SGTA as candidate proteins that could potentially be recruited into pathological aggregates in neurodegenerative disease. We chose CLINT1 and SGTA because intriguingly their homologs (Sgt2p and Ent2p, respectively) were also identified in our yeast model. The Hsp70 protein HSPA8 was selected because Hsp70s have been previously suspected to play critical roles in neurodegenerative disease [65–67]. We confirmed by immuno-blotting the presence of all three proteins in the highly-purified Htt-Q74-GFP aggregates from PC-12 cells (Fig 5b).

### Confirmation of recruitment of identified proteins to polyQ aggregates by immunocytochemistry

The localization of selected polyQ aggregate-associated proteins was also confirmed by confocal microscopy (Fig 5C). The proteins CLINT1, RAD23B and FUS were selected because they appeared to be particularly strong hits based on the initial analysis of the polyQ mass spectrometry data and Western blot results. Using immunocytochemistry, each was observed to be aberrantly recruited to the major sites of HttQ74-GFP aggregation (Fig 5C). However, despite strong over-expression of HttQ74-GFP and the formation of large intracellular aggregates, the recruited proteins did not appear to completely localize to the aggregates. In fact, only a fraction of each protein’s respective total was found at the aggregate.

### Intrinsically-disordered domains play a role in the recruitment of proteins to polyQ aggregates

We selected yeast Sgt2p and human FUS to further examine the role of ID domains in localization to Htt-polyQ aggregates. Sgt2p is involved in protein quality control and does not contain a known RNA-binding domain (RBD). Interestingly, we also identified Sgt2p’s mammalian homolog, SGTA, among the proteins associated with polyQ aggregates in PC-12 cells. The FUS protein contains a distinct RBD and a long N-terminal ID domain, and when expressed in yeast, exhibits aggregation and toxicity reminiscent of what is observed in diseased motor neurons [68–71].

**Fig 6:**
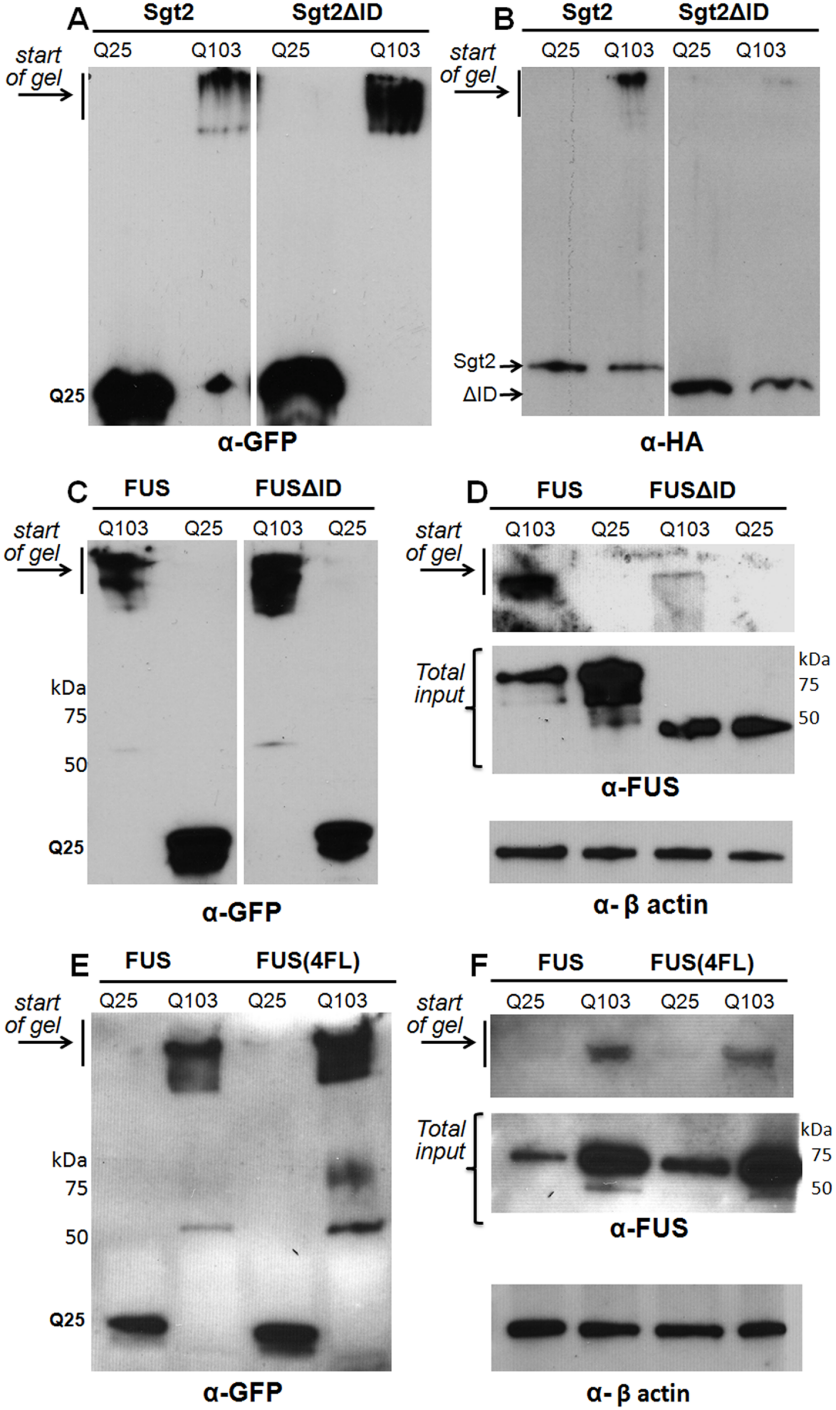
The ID domains of Sgt2p and Fus mediate their localization to Htt-polyQ aggregates in yeast cells. (A, B) Western blots of lysates from yeast strain W303 expressing HttQ25-GFP or HttQ103-GFP in combination with HA-tagged Sgt2p or Sgt2ΔID (A = αGFP; B = αHA). (C, D) Western blots of cells expressing HttQ25-GFP or HttQ103-GFP in combination with FUS or FUSΔID (C = αGFP; D = FUS & α-β actin). Because FUS is quickly degraded in non-denaturing conditions, input controls using urea lysis of cells were included to show initial protein loads. (E, F) Western blots of FUS or FUS(4FL) in HttQ25-GFP-expressing or HttQ103-GFP-expressing cells.

To determine the contribution of their respective ID domains toward recruitment to polyQ aggregates, we created expression vectors in which the major ID domain (as determined by IUPred-L) of both FUS and Sgt2p is deleted (FUSΔID = FUSΔ^1–134^; Sgt2ΔID = Sgt2Δ^300–346^). The full-length versions of FUS and Sgt2p, or their ΔID counterparts, were co-expressed with either HttQ25-GFP or HttQ103-GFP. We truncated the TAPI protocol to easily evaluate the co-localization of the proteins with polyQ aggregates (lysate partitioning; see methods). When we isolated the Htt-polyQ aggregates by lysate partitioning from the Sgt2-transformed strains (shown in Fig 6A as the resistant species stuck at the top of the gel), we observed an enrichment of full-length Sgt2p in the Htt-polyQ high molecular weight aggregates (Fig 6B, left panel). However, when the major ID domain was deleted, most of the co-localization with the polyQ aggregate is eliminated (Fig 6B, right panel). The exact same pattern was observed for FUS and FUSΔID (Fig 6C and 6D, respectively). For comparison, we also used an engineered variant of FUS, which has leucines in place of four conserved phenylalanines (FUS(4F-L): amino acids 305, 341, 359 and 368) in the RNA recognition motif (RRM) domain. FUS(4F-L) was previously shown to be RNA-binding incompetent [72]. We observed that FUS(4F-L) was recruited to polyQ aggregates as readily as wild-type FUS (Fig 6E and 6F), thus suggesting that RNA binding may not play a significant role in facilitating a protein’s inclusion into polyQ aggregates.

## Discussion

### Compositional analysis of polyQ aggregates using TAPI

Models of huntingtin exon 1 mimic truncated versions of huntingtin found in intraneuronal aggregates [73], and thus are helpful for studying intracellular aggregation. Here we analyze the protein species that get recruited into amyloid-like aggregates formed by polyQ-expanded Huntingtin exon 1 in both yeast (Q103) and mammalian cells (Q74). The distinctive tinctorial properties and detergent resistance of the aggregates indicates that they are in an amyloid-like state (S1B Fig).

Proteins that specifically associate with polyQ aggregates are hypothesized to play either positive or negative roles in pathogenic processes. Previous attempts to identify the proteins that interact with huntingtin, have employed yeast-two-hybrid, immuno-precipitation and immuno-histochemical screening [27, 30, 32, 33, 74–78]. The overlap in identified proteins from different methods is often low or the identification of hundreds of proteins limits clarity. Also, antibody-based screening is limited to a predetermined set of functioning antibodies and thus may overlook unexpected interactions. Our mass spectrometry-coupled approach, called TAPI, is specific to the most chemically-resistant forms of protein aggregates and eliminates the need to excise individual bands from acrylamide gels [9, 28]. The high stringency of TAPI—due to DNase, RNase, and detergent treatment along with SDS-gel electrophoresis of aggregates—minimizes the identification of non-specific and loosely-associated proteins. In sum, our data set, in addition to the work of others, helps identify the important factors that make specific proteins most vulnerable to inclusion into polyQ aggregates.

Our analysis in both yeast and mammalian systems revealed a compact group of proteins enriched in polyQ aggregates. In both cell types, the proteins recruited to aggregates belonged to common functional classes (Tables 1 & 2); RNA-binding, endocytosis-related and mitochondrial proteins were disproportionately found in all Htt-polyQ samples. Importantly, the proteins identified by mass spectrometry could be independently confirmed by immuno-blotting. Moreover, our findings are supported by a previous study in Neuro 2A cells, which coupled Sarkosyl treatment with conventional 1D-gel separation and manual band excision, to identify polyQ-associated proteins [67]. Among the twelve proteins identified by Mitsui and coworkers, 8 were also identified by our approach in PC-12 cells (EEF1A1, HSPA8, HSP90AB1, MLF2, PSMC1, RAD23B, UBQLN2, YWHAB; Table 1), suggesting that across cell types, certain proteins consistently have a high propensity for inclusion into polyQ aggregates. Moreover, functional orthologs are common to both the yeast and mammalian samples (YWHAB/Bmh1/2p, DDX5/Dhh1p, SGTA/Sgt2p, CLINT1/Ent1/2p, hnRNPA3/Hrp1 and HSPA8/Ssa1p). The recruitment of SGTA/Sgt2p and CLINT1/Ent2p into polyQ aggregates was confirmed in both the yeast and mammalian systems by immuno-detection methods. This overlap—not only between TAPI samples, but also across divergent organisms—suggests that these specific proteins (or their properties) may play a role in processes linked to pathological polyQ aggregation. This is supported in the literature where Ssa1p [35], HSP70 [79] and Sgt2p [77] have all been directly connected to polyQ aggregates in various models. As for the clathrin-interacting proteins CLINT1 and Ent2p, their presence suggests that aggregates can have important interactions with vesicle-dependent processes.

### RNA binding proteins are disproportionately recruited to polyQ aggregates

RNA-binding proteins were highly represented in the Htt-polyQ TAPI data sets from yeast and rat cells. The presence of quality-control proteins, such as chaperones, was not particularly surprising since Htt-polyQ forms toxic intracellular aggregates. However, the enrichment of RNA-binding proteins was unexpected, especially considering the extensive nuclease treatment that is used prior to isolation of the aggregates (S1B Fig). RNA-binding proteins have been shown to contribute to the pathologies of a number of neurodegenerative diseases [55, 80]. The aggregation of RNA-binding proteins is transient in normal cellular homeostasis, but may accumulate in neurodegenerative diseases due to pathological alteration of assembly and clearance pathways [55]. This aberrant accumulation is frequently tied to interactions mediated by “prion-like” domains [81, 82], which are intrinsically unstructured domains that resemble the domains that enable certain yeast proteins to adopt self-propagating amyloid conformations. It is this intrinsically-disordered property that is likely responsible for the abundance of RNA-binding proteins in polyQ aggregates, because nucleic acid-interacting proteins frequently having intrinsically unstructured regions. Thus, our results suggest that a proteins’s ID domain, not the RNA binding per se, may be the major determinant of inclusion into Htt-polyQ aggregates (Fig 6C and 6D). This is corroborated by our observation that disrupting the RNA-binding domain of FUS had no effect on its inclusion in polyQ aggregates (Fig 6E and 6F).

### Mitochondrial proteins are found in polyQ aggregates

Mitochondrial proteins represent a significant fraction of the polyQ-associated proteins in yeast and rat cells, 8% and 18% respectively. In yeast, this percentage is less than the proteome representation of mitochondrial proteins (~18[83]), but in rat cells this is an over-representation (5–12%[84]). In yeast cells the polyQ aggregates form primarily in the cytoplasm, and in the rat cells the aggregates are equally in the nucleus and cytoplasm. Mitochondrial proteins are mostly synthesized in the cytoplasm and transported as unfolded polypeptides into mitochondria post-translationally. A probable explanation for the presence of mitochondrial proteins in aggregates is that because of their unfolded conformation they are vulnerable to integration into aggregates or because of their dependence on chaperones may be more sensitive to general problems associated with protein quality control.

### Intrinsically-disordered domains facilitate protein recruitment to polyQ aggregates

Why are proteins with ID domains tightly and disproportionately associated with Htt-polyQ aggregates? Not only did we observe an enrichment of ID domain-containing proteins, but previous proteomic studies of polyQ also reveal data sets that are rich in such proteins [32, 78, 85]. Ratovitski *et al*. observed that proteins with ID domains of ≥30 amino acids were enriched in aggregates formed by Htt-50Q in HEK293 cells. This is consistent with our results, although we found significant enrichment with very long ID domains (≥100aa). Similarly, Raychaudhuri *et al* performed a bioinformatic analysis of intrinsic disorder in neurodegenerative disease-associated proteins. Their bioinformatics dataset (obtained from Entrez Gene database keyword search) when compared to control datasets indicates an increase in intrinsic disorder (using FoldIndex) for Huntington disease-associated proteins with ID domains up to 100 amino acids in length [86]. Intrinsically unstructured regions frequently facilitate molecular interactions or serve as sites of post-translational modifications [60], as well as being prominent features of many nucleic acid-binding proteins and chaperones [87, 88]. These domains generally lack hydrophobic residues sufficient for adopting a folded structure in aqueous environment [89] and thus may be more accessible to aggregation simply by virtue of accessibility. We demonstrated that elimination of the major ID domains of two proteins eliminated their co-aggregation with polyQ (Fig 5).

While we do not assert that ID domains are solely responsible for association with Htt-polyQ aggregates, it is clear that ID domain content plays a prominent role in recruiting secondary proteins to aggregates. In some cases, quality-control proteins could even employ intrinsic disorder within a specific domain to facilitate the functional recognition of misfolded protein aggregates. However, because of the large number of quality-control proteins that we identified it cannot be concluded that there is a single mechanism by which all such proteins are tightly associated with polyQ aggregates; some proteins, such as UBQLN2, likely only have specific affinity for aggregates following ubiquitination.

### Why are neurodegenerative disease-associated proteins recruited to polyQ aggregates?

Neurodegenerative disease-linked proteins that have been identified in cellular inclusions in their respective diseases represent a fifth (19/91) of the total TAPI-identified Htt-polyQ aggregate-associated proteins (Table 3). Examining this sub-set reveals that nearly all of them contain ID domains. We observed that some ALS-associated proteins were trapped in polyQ aggregates. Three of these proteins—FUS, HNRPA1 and TDP-43 –are RNA-binding proteins that form pathological inclusions in certain forms of ALS and frontotemporal lobar dementia (FTLD) [90–92]. These proteins have ID domains that resemble the domains of yeast prion proteins due to similar amino-acid composition. It has been concluded that these prion-like domains may be primary-aggregating species, but the fact that these ALS-associated proteins are pulled into polyQ aggregates suggests that in some long-lived cells, such as neurons, there could be underlying protein quality control problems with proteins like FUS and TDP-43 getting preferentially recruited into pre-existing primary aggregates [93]. For example, it is possible that over decades, intermediate-length polyQ expansions in various proteins lead to persistent aggregates that recruit proteins with ID domains; these inclusions would be marked (*i*.*e*. immuno-positive) by specific aggregation-prone proteins. Alternatively, the diminution of protein-quality control with aging may create a cellular environment where proteins with long ID domains are increasingly susceptible to aggregation, thus polyQ models and their induced stress may be good tools for identifying proteins that are most vulnerable to aggregation (or functionally localize to aggregates) under conditions of compromised protein-quality control. We hypothesize SGTA and CLINT1—proteins we confirmed to co-aggregate with polyQ—may be two such candidate proteins with the potential to localize to multiple types of pathological neuronal inclusions. However, just because proteins co-localize with polyQ aggregates in cell models does not mean they will similarly co-localize in animal models or in diseased neurons. HSPA8, whose presence we confirmed in polyQ aggregates in PC-12 cells was not observed in cytoplasmic polyQ aggregates in a transgenic mouse model of Huntington’s disease [94].

### Mechanisms of toxicity

Both the yeast and mammalian model systems show a correlation between polyQ-expanded huntingtin aggregation and cellular toxicity [35, 39], however much debate still persists as to the mechanism by which aggregation leads to cell death [95]. One possible mechanism is that sequestration of proteins to an aggregate may impair cellular function [96], which is arguably an indirect loss of function. Such sequestration by aggregates of polyQ-expanded Ataxin3 (spinocerebellar ataxia-causing protein) was proposed to cause a loss-of-function toxicity [26]. Our observations suggest this indirect loss-of-function toxicity due to sequestration of essential proteins could occur with huntingtin aggregation as well. We observe altered cellular localization for a subset of proteins when Htt-polyQ aggregates are present. It is possible that these proteins may maintain some function while associated with the Htt-polyQ aggregate. If the ID domain of a protein becomes embedded in the polyQ aggregate, while globular or functional domains remain peripheral, function in the wrong place at the wrong time could be a gain-of-function toxicity associated with aggregates. Stoichiometrically, this may be more plausible than loss-of-function because we observe only a fraction of any given co-aggregating species mis-localized to the polyQ aggregate, which itself is quite abundant due to over-expression of Htt (Fig 5). Of course, a combination of gain-of-function and loss-of-function mechanisms could contribute to the overall cellular toxicity.

Although many techniques have been employed to identify huntingtin-interacting proteins, few examine specifically the amyloid form. Our results show that a select group of proteins are trapped by polyQ amyloid-like aggregates. Proteins with long ID domains are disproportionately prone to inclusion, as are many proteins that are associated with other neurodegenerative diseases. The enrichment in ID domain-containing proteins in polyQ aggregates, and the elimination of this enrichment when the ID domains are deleted, reveals the significant role of protein structure in determining if a protein gets secondarily recruited into certain types of aggregates. Thus, while some proteins might be predicted to be recruited into aggregates because of their function (*i*.*e*. quality-control proteins or proteins that interact with the soluble form of an aggregating species), many proteins may be recruited simply as a consequence of their secondary and tertiary structural elements. The metastable structure and accessibility of long ID domains may render proteins particularly susceptible to aberrant inclusion in amyloid-like aggregates. Recently, Habch and colleagues put forth the idea that ID proteins represent a class of pharmacological targets [97]. As our results suggest, if the recruitment of specific ID domain-containing proteins into pathological aggregates is critical to cellular degeneration, then targeting ID domains to reduce their sequestration may have therapeutic potential in a variety of neurodegenerative diseases.

## Supporting Information

S1 Fig Additional controls for TAPI method. A—Comparison of protein size and abundance for TAPI proteins versus the yeast proteome. B—Efficacy of Rnase treatment in the TAPI procedure (left panel) and Th-T fluorescence of crude aggregates isolated from yeast expressing Htt-Q103-GFP or HttQ25-GFP (right panel). C—Proteasomal inhibition or proteo-toxic stress is not sufficient to cause Sgt2p to be trapped in (or form) detergent-resistant high-molecular weight aggregates.
(PDF)

S1 File Proteins identified by mass spectrometry following TAPI purification of polyglutamine aggregates from yeast cells. This Excel file contains tables that list initial mass spectrometry results for multiple yeast samples and the values for ID domains for all identified proteins, as well as controls. S1A includes the proteins identified by our stringent binary analysis (see methods) and contains biochemical characterization of the identified proteins. S1B contains an expanded list of identified proteins using a less stringent arbitrary threshold of the mass spec data. S1C includes the list of 100 random yeast proteins used for comparison purposes. S1D includes the RBPs identified by TAPI as well as the proteomic RBPs, identified by Gene Ontology search, and their ID domains as predicted by IUPred-L.
(XLSX)

S2 File Proteins identified by mass spectrometry following TAPI purification of polyglutamine aggregates from rat cells. This Excel file contains tables that list initial mass spectrometry results for multiple rat samples and the values for ID domains for all identified proteins, as well as controls. S2A includes the proteins identified by the stringent binary analysis (see methods) and contains biochemical characterization of the identified proteins. S2B is an expanded list of identified proteins using a less stringent arbitrary threshold of the mass spec data. S2C includes the list of 200 random rat proteins and their ID domains as predicted by IUPred-L.
(XLSX)

S3 File PERL-based algorithm for examining protein sequences for Q/N-rich regions. (PL)

S1 Table Analogous proteins in yeast and rat associate with Htt-PolyQ aggregates. (DOCX)
