# Supplementary material for: Proteins with Intrinsically Disordered Domains Are Preferentially Recruited to Polyglutamine Aggregates
Source: PLoS One. 2015 Aug 28;10(8):e0136362. doi: 10.1371/journal.pone.0136362 (PMC4552826; doi:10.1371/journal.pone.0136362)

Supplemental Figure S1A: Comparison of protein size and abundance. The yeast proteome is compared with the 52 proteins identified by TAPI as being tightly associated with Htt-Q103-GFP. The proteins found to co-aggregate with polyQ were not obviously larger or more abundant than the yeast proteome in general (TAPI avg. 1.2×10^4 copies/cell, Proteome avg. 1.3×10^4 copies/cell). TAPI does not enrich for disproportionately large proteins (Avg TAPI protein size 72kDa, proteome avg 53kDa). Protein abundance values accumulated from western blotting (Ghaemmaghami et al., Nature 2003), GFP expression (Newman et al., Nature 2006), 2D gel analysis (Futcher et al., Mol Cell Biol 1999), and APEX analysis (Lu et al., Nat. Biotechnol. 2007).

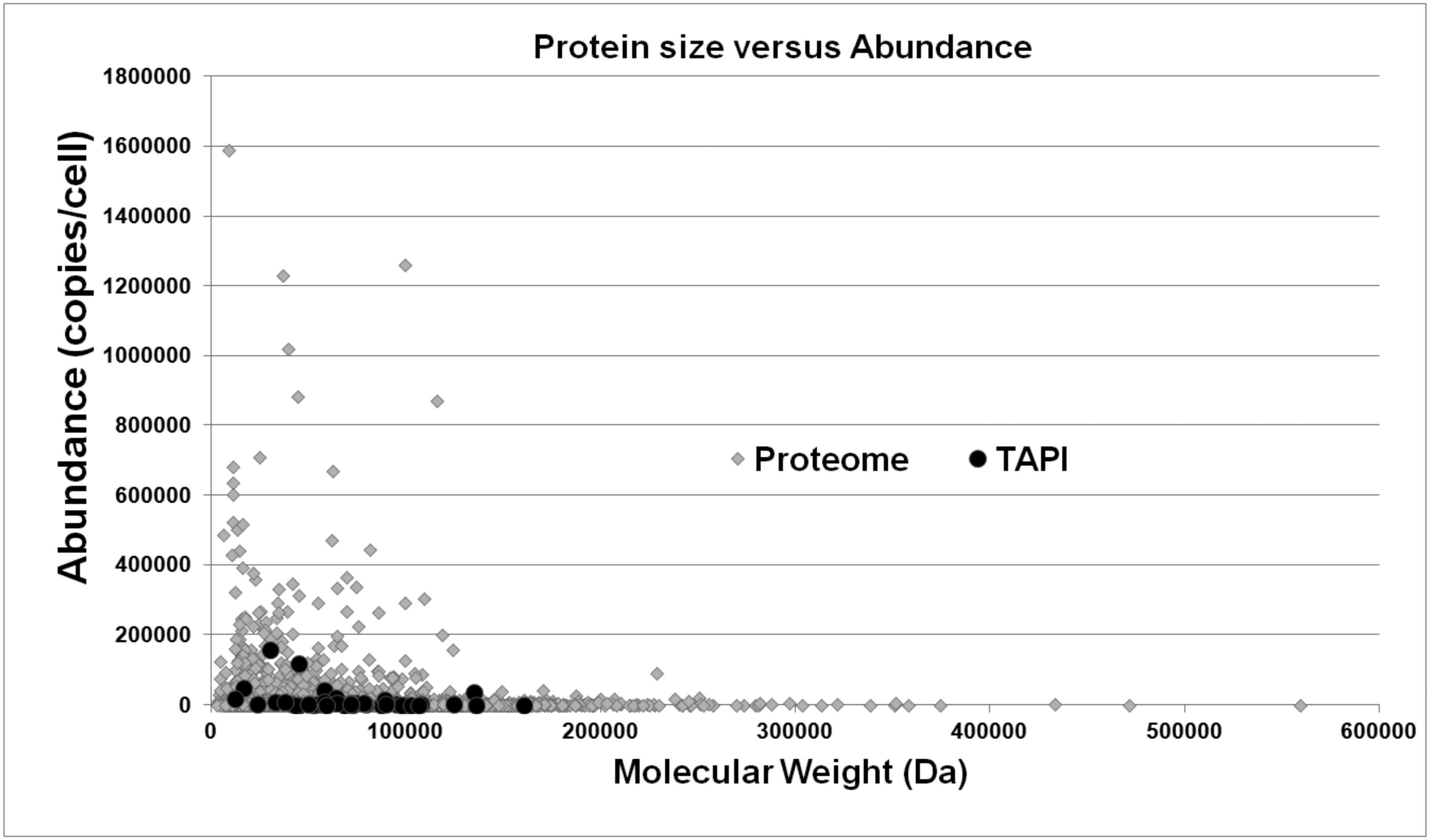

Supplemental Figure S1B: Left panel – Efficacy of Rnase treatment in TAPI procedure. **Right panel –**Th-T fluorescence of crude aggregates isolated from yeast expressing Htt-Q103-GFP or HttQ25-GFP.

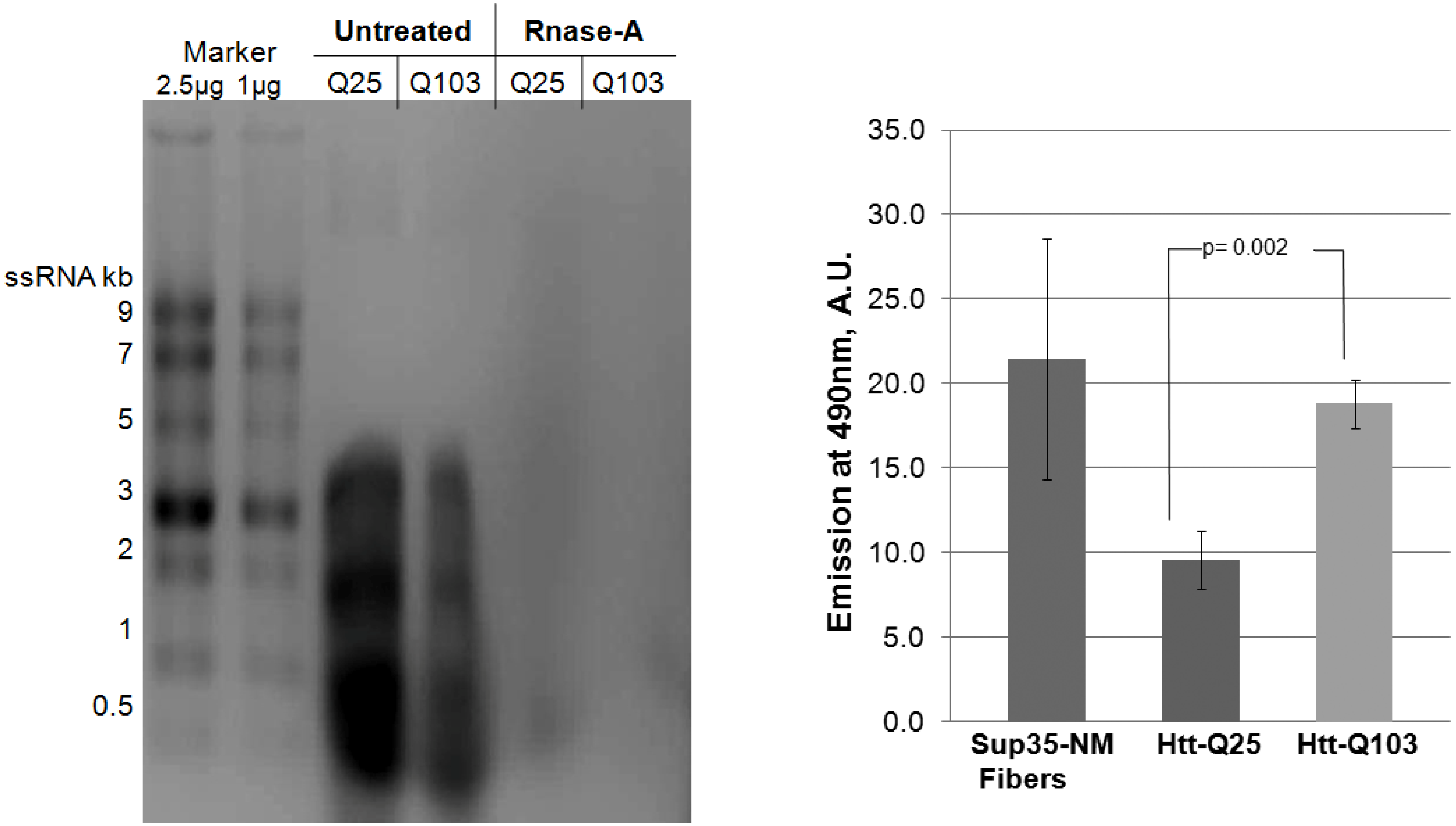

Supplemental Figure S1C: Proteasomal inhibition or proteo-toxic stress are not sufficient to cause Sgt2p to be trapped in (or form) detergent-resistant high-molecular weight aggregates. Neither the overnight exposure (~16 hours) of yeast cells to 10 µM MG-132 nor the overnight expression of human alpha-synuclein altered the electrophoretic migration of yeast Sgt2p. However, the accumulation of Q103-GFP into a large detergent-resistant species was sufficient to affect Sgt2p's migration into an acryalmide gel.

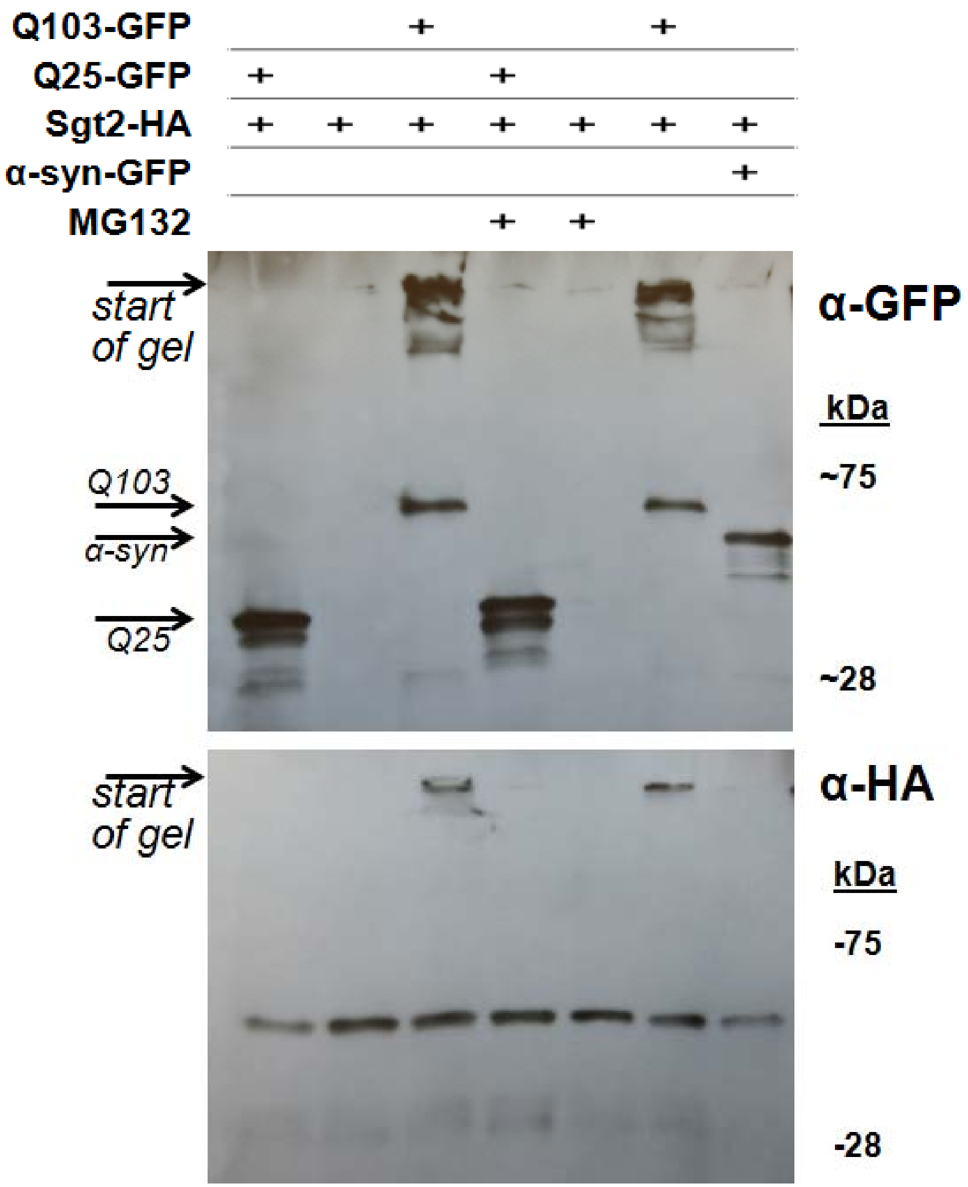

Supplement: S1 Fig — A—Comparison of protein size and abundance for TAPI proteins versus the yeast proteome. B—Efficacy of Rnase treatment in the TAPI procedure (left panel) and Th-T fluorescence of crude aggregates isolated from yeast expressing Htt-Q103-GFP or HttQ25-GFP (right panel). C—Proteasomal inhibition or proteo-toxic stress is not sufficient to cause Sgt2p to be trapped in (or form) detergent-resistant high-molecular weight aggregates. (PDF) [file pone.0136362.s001.pdf]
