# Supplementary material for: Proteins with Intrinsically Disordered Domains Are Preferentially Recruited to Polyglutamine Aggregates
Source: PLoS One. 2015 Aug 28;10(8):e0136362. doi: 10.1371/journal.pone.0136362 (PMC4552826; doi:10.1371/journal.pone.0136362)
Supplement: S1 Table — (DOCX) [file pone.0136362.s005.docx]

| **Rat TAPI Protein** | **Yeast TAPI protein** | **Function** |  |
| --- | --- | --- | --- |
| Aak1 | Akl1 | Ser/Thr kinases involved in endocytosis and actin cytoskeleton organization; recruit endocytic accessory factors |  |
| Sfn/Ywhab | Bmh1 | 14-3-3 proteins; associated with diverse protein binding and signaling activities |  |
| Dnaja2/Dnaja4/  Dnajc7/Dnajb1 | Ydj1/Apj1 | HSP40 co-chaperones; function with HSP70s; generally involved in protein folding and quality control |  |
| Hnrnpa3 | Hrp1 | Heterogeneous nuclear ribonucleoproteins; bind RNA; involved in RNA processing |  |
| Sgta | Sgt2 | Glutamine-rich cytoplasmic co-chaperone; functions in post-translational membrane insertion of proteins in yeast |  |
| Clint1 | Ent1/Ent2 | Epsin-like proteins involved in endocytosis; clathrin interactors |  |
| Ddx5 | Dhh1 | Cytoplasmic DExD/H-box RNA helicases; multiple RNA-related functions |  |
|  | | |  |

**Supplementary Table 1**: Analogous proteins from both yeast and rat cells associate with Htt-PolyQ aggregates.

Protein similarity determined using Ensembl comparative genomics, RGD, SGD, and % identity.
